# Does caffeine intake enhance physical and physiological performance in swimmers? a systematic review and meta-analysis

**DOI:** 10.3389/fphys.2025.1648862

**Published:** 2025-12-05

**Authors:** Dongxiang Huang, Bo Huang, Zhongzheng Wang, Hideki Takagi, Yang Chen, Zhuonan Huang, Xiaobing Wang, Xiangyue Tong

**Affiliations:** 1 School of Physical Education, Shaoguan University, Shaoguan, China; 2 School of Physical Education and Sports Science, South China Normal University, Guangzhou, China; 3 Faculty of Health and Sport Sciences, University of Tsukuba, Ibaraki, Japan; 4 Department of Physical Education, Guangdong University of Foreign Studies, Guangzhou, China; 5 Office of Academic Affairs, Guangdong Vocational Institute of Public Administration, Guangzhou, China

**Keywords:** sports nutrition, swimmers, caffeine, swimming performance, meta-analysis

## Abstract

**Background:**

Acute caffeine intake is widely used by athletes to enhance performance, and its ergogenic effects are well-established in several land-based sports. However, whether similar benefits apply to swimming remains unclear.

**Objective:**

This systematic review and meta-analysis (SRMA) evaluates the effects of acute caffeine intake on swimmers’ physical and physiological performance.

**Methods:**

Following PRISMA guidelines, studies were retrieved from five databases: Cochrane Library, Web of Science, Embase, PubMed, and SPORTDiscus. Eligible trials assessed acute caffeine effects on physical (25 m time, 50 m time, swimming velocity, jump height, power) and physiological (blood lactate, blood pH) outcomes in swimmers. A random-effects model was used to compute standardized mean differences (SMD) and 95% confidence intervals (CI). Subgroup analyses were conducted based on the type of outcome measures used.

**Results:**

A total of ten randomized crossover trials involving 121 participants were included. Acute caffeine intake showed no significant improvement in 25 m time (SMD = −0.14; 95% CI: -0.91, 0.63; p = 0.73), 50 m time (SMD = −0.06; 95% CI: -0.50, 0.37; p = 0.78), swimming velocity (SMD = 0.47; 95% CI: -1.97, 2.92; p = 0.70), jump height (SMD = 0.12; 95% CI: -0.72, 0.96; p = 0.78) and power (SMD = 0.05; 95% CI: -0.78, 0.89; p = 0.90). Additionally, no significant effects were observed for blood lactate (SMD = 0.81; 95% CI: -0.07, 1.70; p = 0.07), and blood pH (SMD = 0.01; 95% CI: -1.86, 1.88; p = 0.99).

**Conclusion:**

This SRMA found no significant evidence that acute caffeine supplementation improves swimming performance, either physical or physiological. Given the small number and sample sizes of included studies and the presence of heterogeneity, the findings should be regarded as preliminary. Larger, well-designed trials are needed to clarify caffeine’s true effects on swimmers’ performance and to inform practical recommendations.

## 1 Introduction

Swimming is a highly competitive sport in which even the slightest time differences can determine the outcome, particularly in sprint events. For instance, the gap between first and second place in the men’s and women’s 50 m freestyle events has been reported to be as narrow as 0.01 s and 0.02 s, respectively (Kwok et al., 2021). Given such fine margins, athletes, coaches, and researchers continuously explore potential methods for optimizing swimming performance. Among these, dietary supplements have attracted increasing attention for their potential role in optimizing athletic performance (Huang et al., 2024a).

Among dietary supplements, caffeine is one of the most thoroughly investigated substances in sports science. The International Olympic Committee (IOC) has recognized caffeine as a legal nutritional aid, while the International Society of Sports Nutrition (ISSN) classifies it as a supplement with substantial evidence supporting its effects in various sports contexts (Kerksick et al., 2018). Consequently, caffeine consumption is prevalent among athletes, including swimmers (Van Thuyne and Delbeke, 2006). Mechanistically, evidence indicates that caffeine has an effect on the central nervous system, potentially enhancing motor neuron activation and mitigating declines in voluntary activation induced by fatigue (Bazzucchi et al., 2011; Cristina-Souza et al., 2022). Additionally, some studies suggest that caffeine may elevate blood lactate levels without a corresponding increase in perceived exertion, which could, in theory, affect high-intensity, short-duration efforts (Goods et al., 2017). These potential physiological effects have led to growing interest in the role of caffeine intake in swimming performance.

Despite these theoretical mechanisms, the influence of caffeine consumption on swimming performance remains inconclusive. While some studies have reported improvements in swim performance following caffeine ingestion (Acar et al., 2024; Collomp et al., 1992; Goods et al., 2017; Lara et al., 2015; MacIntosh and Wright, 1995; Salgueiro et al., 2022; Vanata et al., 2014), others have found no discernible effect (Alkatan, 2020; Newbury et al., 2022; Pruscino et al., 2008).

A previous meta-analysis by Grgic (2022) reported that caffeine supplementation had a positive effect on swimming performance, but this analysis presented notable limitations (Grgic, 2022). Specifically, Grgic (2022) pooled data from swim trials ranging from 25 m to 75 m without distinguishing between race distances, which may have introduced confounding effects and masked distance-specific responses. In addition, Grgic (2022) failed to examine physiological markers that may reflect the metabolic effects of caffeine, such as blood lactate and blood pH. Blood lactate is a well-established marker of anaerobic metabolism (Gladden, 2004; Beneke et al., 2011; Brooks, 2001), while blood pH reflects acid-base balance, and the two are closely linked during high-intensity exercise (Robergs et al., 2004). Although changes in these measures may not directly determine performance outcomes, particularly in short-distance events such as 25 m and 50 m, they provide valuable insights into the mechanisms through which caffeine may influence exercise metabolism. Moreover, jump height and power, indirect yet relevant indicators of physical performance, were also not considered. Finally, Grgic (2022) was restricted to literature published before 2021 (Grgic, 2022). Since then, three additional studies (Acar et al., 2024; Newbury et al., 2022; Salgueiro et al., 2022) have been published, highlighting the need for an updated and more comprehensive analysis.

To overcome these limitations, this systematic review and meta-analysis (SRMA) seeks to deliver an up-to-date and comprehensive assessment of the effects of acute caffeine intake on swimming performance. Specifically, we (1) stratify outcomes by swim distance, (2) include both direct indicators of physical performance (e.g., 25 m time, 50 m time, swimming velocity) and indirect proxies (e.g., jump height, power), (3) evaluate physiological biomarkers (e.g., blood lactate, blood pH), and (4) incorporate all relevant studies published through 2025. This enhanced analytical framework enables a more precise and timely synthesis of the available evidence, offering new insights into the role of caffeine in modulating both physical and physiological aspects of swimmer performance.

## 2 Methods

This SRMA, performed in line with PRISMA guidelines (Page et al., 2021), investigated the impact of caffeine on physical and physiological performance of swimmers. The PRISMA 2020 Checklist is provided in Supplementary Table S1. The study was pre-registered on PROSPERO https://www.crd.york.ac.uk/PROSPERO/view/CRD42025642300.

### 2.1 Literature search strategy

Two authors (D.X.H. and Z.Z.W) independently conducted the literature search, with discrepancies resolved by a third reviewer (X.B.W.). Relevant studies were identified across five major databases, including Cochrane Library, Web of Science, Embase, PubMed, and SPORTDiscus, from inception to 25 January 2025. The search strategy employed Boolean terms: (caffeine OR coffee OR “energy drink”) AND (swimming OR swimmer* OR swim), with details presented in Supplementary Table S2. Based on the PICOS framework outlined in the PROSPERO registration, the initial search focused on the effects of caffeine use by swimmers. However, upon reviewing the literature, all identified studies specifically addressed the acute effects of caffeine supplementation, prompting us to limit our analysis to this subset of studies.

### 2.2 Inclusion and exclusion criteria

Studies were chosen in accordance with the PICOS framework. Population (P): Studies were required to include healthy swimmers, regardless of age, sex, or training level. Intervention (I): Studies examining the isolated effects of acute caffeine supplementation, regardless of its form, were included. Comparator (C): A placebo-controlled design was required for inclusion. Outcomes (O): Studies were required to assess at least one performance-related outcome, including direct physical measures (e.g., 25 m time, 50 m time, swimming velocity), indirect indicators (jump height, power), or physiological biomarkers (e.g., blood lactate, blood pH). Study Design (S): Inclusion was limited to randomized, crossover, placebo-controlled trials. Only peer-reviewed original research articles published in English were considered. Blinding procedures were not considered a strict inclusion criterion. Instead, information on blinding was extracted when available and incorporated into the assessment of methodological quality and risk of bias.

Studies involving athletes from other aquatic disciplines (e.g., triathletes, water polo players, synchronized swimmers, or paraplegic swimmers) were excluded. Studies incorporating caffeine alongside other supplements were excluded unless the distinct effects of caffeine and placebo could be clearly delineated. Studies lacking a placebo group or a controlled comparison were excluded. Non-original publications (e.g., editorials, reviews, commentaries, and conference proceedings) were not considered. Exclusions were determined solely on the basis of the predefined PICOS criteria, and study quality ratings (e.g., PEDro scores) were not used as exclusion thresholds.

### 2.3 Text screening

Two authors (D.X.H. and Z.Z.W.) independently screened titles and abstracts using a customized Excel screening form, followed by independent full-text assessments. Disagreements were resolved by discussion or consultation with a third author (X.B.W.). Inter-rater reliability was quantified using Cohen’s Kappa (Landis and Koch, 1977).

### 2.4 Data extraction

Two authors independently extracted data using a pre-designed standardized Excel form, which was piloted on a subset of studies to ensure clarity and consistency (D.X.H. and Z.Z.W.), with discrepancies resolved by a third author (X.B.W.). Inter-rater reliability between the two reviewers was quantified using Cohen’s Kappa (Landis and Koch, 1977). Extracted data included: (i) study details (e.g., countries and study design), (ii) population details (e.g., status, sample size, gender, age), (iii) intervention details (e.g., time of ingestion, dosage, duration, form), and (iv) outcomes (e.g., 25 m time, 50 m time, swimming velocity, jump height, power, blood lactate, blood pH). Data presented graphically were extracted with Web Plot Digitizer V4.0 (Free Software Foundation, Boston, MA, United States) (Drevon et al., 2017). For studies with missing essential data, we intended to contact the corresponding authors to request the missing information. However, despite our efforts, we were unable to establish contact with the corresponding authors of two studies (MacIntosh and Wright, 1995; Pruscino et al., 2008) and, as a result, were unable to obtain the missing data. This is reflected in the missing data presented in Table 1. Standard errors presented in some studies were transformed into their corresponding standard deviations.

**TABLE 1:** Summary of the included studies.

No	Study details			Population details				Intervention details				Outcomes	Condition
	Study	Countries	Study design	Status	Sample size	Gender	Age (year)	Time of ingestion	Dose	Duration (days)	Form		
1	Acar et al. (2024)	Turkey	Randomized Double-blind, crossover	Competitive swimmers	8	F: 8	21.3 ± 1.4	60 min before the trials	6 mg/kg/bm	2	Capsules	25 m time 50 m time, jump height, power	Non-fatiguing; non-competitive experimental setting
2	Alkatan (2020)	Kuwait	Randomized Double-blind, crossover	Trained swimmers	18	M:18	18–25	45 min before the trials	250 mg	2	Capsules	25 m time	Non-fatiguing; non-competitive experimental setting
3	Collomp et al. (1992)	France	Randomized Crossover	Competitive swimmers	7	M: 3 F: 4	17 ± 2.1	60 min before the trials	250 mg	2	Capsules	Swimming velocity, blood lactate	Non-fatiguing; non-competitive experimental setting
4	Goods et al. (2017)	Australia	Randomized Single-blind, crossover	Competitive swimmers	9	M: 9	20.8 ± 2.8	60 min before the trials	3 mg/kg/bm	2	Capsules	Blood lactate, blood pH	Non-fatiguing; non-competitive experimental setting
5	Lara et al. (2015)	Spain	Randomized Double-blind, crossover	Competitive swimmers	14	M:14	20.2 ± 2.6	60 min before the trials	3 mg/kg/bm	2	Powder	Jump height, power 50 m time Blood lactate	Non-fatiguing; non-competitive experimental setting
6	MacIntosh and Wright (1995)	Canada	Randomized Double-blind, crossover	Competitive swimmers	11	M:7 F:4	21.8 ± 0.5	150 min before the trials	6 mg/kg/bm	2	--	Blood lactate	Non-fatiguing; non-competitive experimental setting
7	Newbury et al. (2022)	UK	Randomized Double-blind, crossover	Competitive swimmers	8	M: 5 F: 3	16–19	60 min before the trials	3 mg/kg/bm	2	Capsules	Blood lactate	Non-fatiguing; non-competitive experimental setting
8	Pruscino et al. (2008)	Australia	Randomized Double-blind, crossover	Competitive swimmers	6	M: 6	--	45 min before the trials	6.2 ± 0.3 mg/kg/bm	4	Capsules	200 m time Blood pH	Non-fatiguing; non-competitive experimental setting
9	Salgueiro et al. (2022)	Brazil	Randomized Double-blind, crossover	Trained swimmers	10	M: 10	18.2 ± 1.7	60 min before the trials	6 mg/kg/bm	2	Capsules	Swimming velocity Blood lactate	Non-fatiguing; non-competitive experimental setting
10	Vanata et al. (2014)	United States of America	Randomized Single-blind, crossover	Competitive swimmers	30	M: 18 F: 12	19.5 ± 1.4	30 min before the trials	3 mg/kg/bm	2	Capsules	50 m time	Non-fatiguing; non-competitive experimental setting

UK: united kingdom, M: male, F: female, min: minute, bm: body mass.

### 2.5 Methodological quality and publication bias

The process was independently carried out by two researchers, D.X.H. and Z.Z.W, with discrepancies resolved by X.B.W. The methodological standards of the selected studies were examined using the Physiotherapy Evidence Database (PEDro) scale (De Morton, 2009). The PEDro scale is widely employed in systematic reviews assessing the efficacy of supplements and nutritional ergogenic aids (Grgic et al., 2020; Mcmahon et al., 2017; Quesnele et al., 2014). It functions as a robust and impartial instrument for evaluating the internal validity of randomized controlled trials (Maher et al., 2003). The scale comprises 11 items, with ratings applied to items 2–11. A score of 1 point is assigned for a positive response, whereas a negative response receives 0 points, yielding a maximum total of 10 points. A higher PEDro score denotes a lower risk of bias, whereas a lower score indicates a greater risk. The PEDro scale quality was categorized as excellent (9–10 points), good (6–8 points), fair (4–5 points), or poor (≤3 points) (Cashin, 2020).

In addition, the Cochrane Risk of Bias 2 (RoB 2) tool was used to assess potential bias in the included randomized controlled trials across five domains: bias from the randomization process, deviations from intended interventions, missing outcome data, measurement of the outcome, and selection of the reported result (Sterne et al., 2019). Each domain was judged as the low risk, some concerns, or high risk. A domain was rated as low risk when the study design and conduct were appropriate, with no indications of bias that could affect the results (e.g., adequate randomization, proper handling of missing data, blinded outcome assessment). Some concerns were assigned when there was limited or unclear information, but potential bias was unlikely to meaningfully alter the results (e.g., incomplete details of randomization, insufficient information on blinding, minor protocol deviations). High risk was assigned when flaws in study design or conduct were likely to introduce bias that could affect the results (e.g., inadequate randomization, major deviations from intended interventions, extensive missing data without appropriate handling). An overall risk-of-bias judgment was then derived for each study according to the RoB 2 decision rules. Overall risk was low if all five domains were judged low risk; some concerns if at least one domain raised some concerns but none was high risk; and high if any single domain was judged high risk or if multiple domains raised some concerns (Sterne et al., 2019). Consistent with RoB 2 guidance, judgments were made at the outcome level, and for each study the overall judgment was summarized based on the primary outcome.

Publication bias was investigated by visually analyzing the funnel plot asymmetry in the combined data. This approach facilitated the evaluation of bias in research dissemination within the review. Formal statistical tests for funnel plot asymmetry (e.g., Egger’s or Begg’s tests) were not performed because fewer than 10 studies contributed to any outcome, and the recommended minimum for ensuring adequate statistical power (Sterne et al., 2011).

### 2.6 Statistical analysis

This SRMA was performed when at least two studies evaluated the same outcome measures. Outcomes reported in only one study were excluded from the quantitative synthesis, as meta-analysis requires pooling results from at least two independent studies to generate a meaningful quantitative estimate (Chandler et al., 2019). Standardized mean differences (SMD) and corresponding 95% confidence intervals (CI) were utilized to examine the effects of caffeine on the physical and physiological performance of swimmers. SMD values were classified based on effect size as follows: trivial (<0.2), small (0.2–0.4), moderate (0.4–0.8), and large (>0.8) (Cohen, 1992). Study heterogeneity was evaluated utilizing the I^2^ statistic, categorized as low (<25%), moderate (25%–75%), or substantial (>75%) (Higgins et al., 2003).

Subgroup analyses were conducted based on the type of outcome measures, categorizing them into physical performance indicators (e.g., 25 m time, 50 m time, swimming velocity, jump height, power) and physiological indicators (e.g., blood lactate, blood pH). A random-effects model was applied to all meta-analyses (DerSimonian and Laird, 1986). A significance level of p < 0.05 was established.

PEDro scores were recorded and summarized using Microsoft Excel (Office 2019). Meta-analyses and funnel plots for publication bias were performed in Review Manager (version 5.4.1; Cochrane Collaboration, United States) (Cumpston et al., 2019). RoB 2 plots were generated in R (version 4.4.2) using the ggplot2 and dplyr packages.

## 3 Results

### 3.1 Search results

**FIGURE 1:**
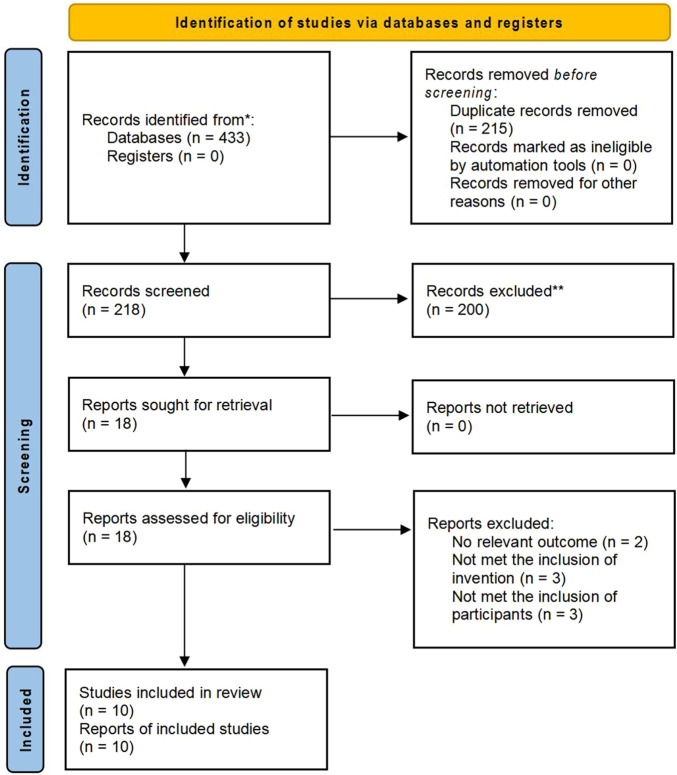
Flowchart of search strategy and article selection process.

An initial search of the database yielded a total of 433 records. Of these, 215 duplicate records were excluded, including 205 identified through EndNote software and 10 manually identified during title and abstract screening. This left 218 unique records for potential inclusion in the SRMA. Screening of titles and abstracts resulted in the removal of 200 studies that did not satisfy the eligibility requirements. Consequently, 18 studies were identified as suitable for full-text review. Of the studies assessed, ten (Acar et al., 2024; Alkatan, 2020; Collomp et al., 1992; Goods et al., 2017; Lara et al., 2015; MacIntosh and Wright, 1995; Newbury et al., 2022; Pruscino et al., 2008; Salgueiro et al., 2022; Vanata et al., 2014) satisfied the inclusion criteria and were retained for the final SRMA. Inter-rater agreement between the two reviewers was substantial (Cohen’s Kappa = 0.72), confirming the reliability of the screening process (Landis and Koch, 1977). Figure 1 presents the flowchart depicting the search strategy employed.

### 3.2 Study characteristics

The inter-rater agreement for data extraction was almost perfect (Cohen’s Kappa = 0.81), confirming the reliability of the extraction process (Landis and Koch, 1977). The ten studies, involving a total of 121 participants, were conducted between Collomp et al. (1992) and Acar et al. (2024), spanning nine countries: Turkey, Kuwait, France, Australia, Spain, Canada, the United Kingdom (UK), Brazil, and the United States of America (United States). Of these, Australia contributed two studies (Goods et al., 2017; Pruscino et al., 2008), while each of the other countries contributed one study. All studies employed a randomized crossover design. The majority of studies involved competitive swimmers as participants, with only two studies reporting trained swimmers (Alkatan, 2020; Salgueiro et al., 2022). Sample sizes varied from six (Pruscino et al., 2008) to 30 (Vanata et al., 2014) participants per study. Most studies focused on male participants (Alkatan, 2020; Goods et al., 2017; Lara et al., 2015; Pruscino et al., 2008; Salgueiro et al., 2022), while four included both male and female swimmers (Collomp et al., 1992; MacIntosh and Wright, 1995; Newbury et al., 2022; Vanata et al., 2014), and one exclusively examined female swimmers (Acar et al., 2024). The studies included in this review utilized varying doses of caffeine, ranging from 3 mg/kg to 6 mg/kg body weight, with caffeine ingestion timing ranging from 30 min (Vanata et al., 2014) to 150 min (MacIntosh and Wright, 1995) prior to the trials. The majority of caffeine was administered in capsule form (Acar et al., 2024; Alkatan, 2020; Collomp et al., 1992; Goods et al., 2017; Newbury et al., 2022; Pruscino et al., 2008; Salgueiro et al., 2022; Vanata et al., 2014), while one study using powder form (Lara et al., 2015). The method of caffeine administration was not specified in one study (MacIntosh and Wright, 1995). The duration of supplementation generally lasted 2 days (Vanata et al., 2014; Salgueiro et al., 2022; Acar et al., 2024; Alkatan, 2020; Collomp et al., 1992; Goods et al., 2017; Lara et al., 2015; MacIntosh and Wright, 1995; Newbury et al., 2022). All studies were conducted in non-fatiguing, non-competitive experimental settings, in which participants were not exposed to prior physical exertion or competitive conditions, thereby ensuring that the observed effects were solely attributable to the intervention. A summary of the study characteristics is provided in Table 1.

### 3.3 Methodological quality and publication bias

With a mean PEDro scale score of 8.6, the included studies exhibited a high standard of methodological rigor. Among the ten studies, six (Acar et al., 2024; Alkatan, 2020; Lara et al., 2015; MacIntosh and Wright, 1995; Salgueiro et al., 2022; Vanata et al., 2014) were rated as excellent quality, while four (Collomp et al., 1992; Goods et al., 2017; Newbury et al., 2022; Pruscino et al., 2008) were deemed to be of good quality. The detailed PEDro scores for each individual study, including item-level ratings and overall quality classification, are presented in Table 2.

**TABLE 2:** Assessment of included studies using the PEDro scale.

Study	1	2	3	4	5	6	7	8	9	10	11	Total
Acar et al. (2024)	Yes	1	1	1	1	1	1	1	1	1	1	10
Alkatan (2020)	Yes	1	1	1	1	1	0	1	1	1	1	9
Collomp et al. (1992)	Yes	1	0	1	0	0	0	1	1	1	1	6
Goods et al. (2017)	Yes	1	1	1	1	0	0	1	1	1	1	8
Lara et al. (2015)	Yes	1	1	1	1	1	1	1	1	1	1	10
MacIntosh and Wright (1995)	Yes	1	1	1	1	1	0	1	1	1	1	9
Newbury et al. (2022)	Yes	1	0	1	1	1	0	1	1	1	1	8
Pruscino et al. (2008)	Yes	1	0	1	1	1	0	1	1	1	1	8
Salgueiro et al. (2022)	Yes	1	1	1	1	1	0	1	1	1	1	9
Vanata et al. (2014)	Yes	1	1	1	1	0	1	1	1	1	1	9

1, Clear eligibility criteria were defined; 2, Volunteers were randomly assigned to the respective groups; 3,Allocation concealment was ensured; 4, Baseline characteristics of the groups were comparable with regard to key prognostic factors; 5, Blinding was applied to all participants; 6, Therapists administering the intervention were blinded; 7, Outcome assessors were blinded; 8, Outcome measures were obtained from at least 85% of the originally allocated participants; 9, All participants with available outcome measures received the assigned treatment or control, or an intention-to-treat analysis was performed; 10, Statistical analyses comparing groups were reported for at least one primary outcome; 11, Point estimates and variability measures were provided for at least one primary outcome.

**FIGURE 2:**
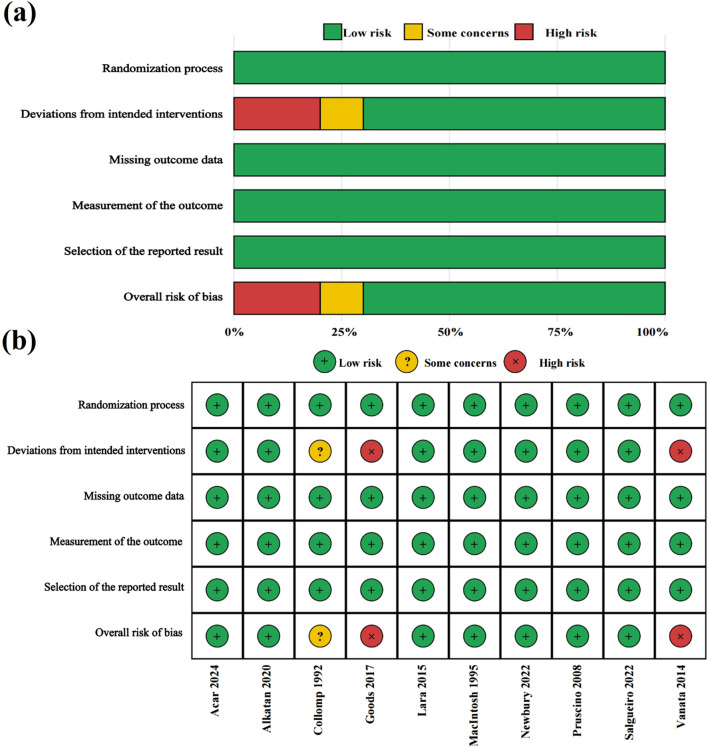
RoB 2 plots: **(a)** domain-level summary and **(b)** traffic-light plot of individual studies.

In addition, the risk of bias was evaluated using the RoB 2 tool. The majority of studies were judged to have a low risk of bias across all five domains (Acar et al., 2024; Alkatan, 2020; Lara et al., 2015; MacIntosh and Wright, 1995; Newbury et al., 2022; Pruscino et al., 2008; Salgueiro et al., 2022). Some concerns were identified in one study (Collomp et al., 1992), whereas two studies (Goods et al., 2017; Vanata et al., 2014) were judged to have a high overall risk of bias. A domain-level summary and a traffic-light plot of individual studies are presented in Figure 2.

**FIGURE 3:**
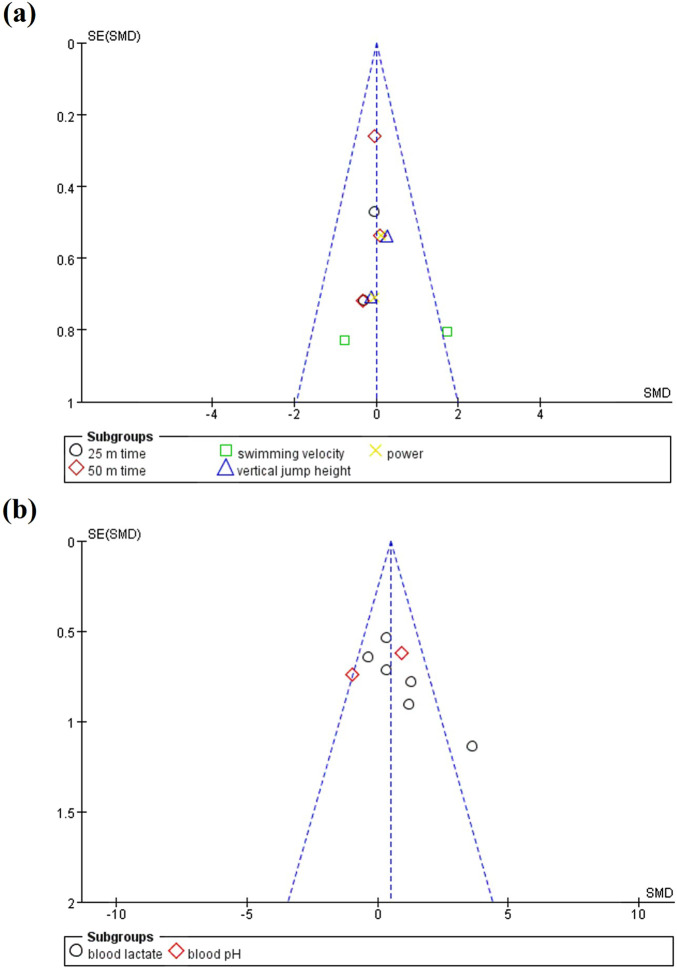
Funnel plots for **(a)** physical performance and **(b)** physiological performance.

Figure 3 illustrates publication bias across various outcomes, including both physical and physiological variables. Figure 3a presents results related to physical performance, such as 25 m time, 50 m time, swimming velocity, power, and vertical jump height. The funnel plot is generally symmetric, with studies evenly distributed around the vertical axis, suggesting no publication bias for these physical performance outcomes. Specifically, the studies for 25 m time, 50 m time, vertical jump height, and power are predominantly clustered near the center, whereas the studies for swimming velocity are more widely distributed. Figure 3b focuses on physiological outcomes, particularly blood lactate and blood pH. The funnel plots for these outcomes exhibit a generally even distribution, indicating no significant publication bias for these results.

### 3.4 Meta-analysis results

#### 3.4.1 Physical performance

**FIGURE 4:**
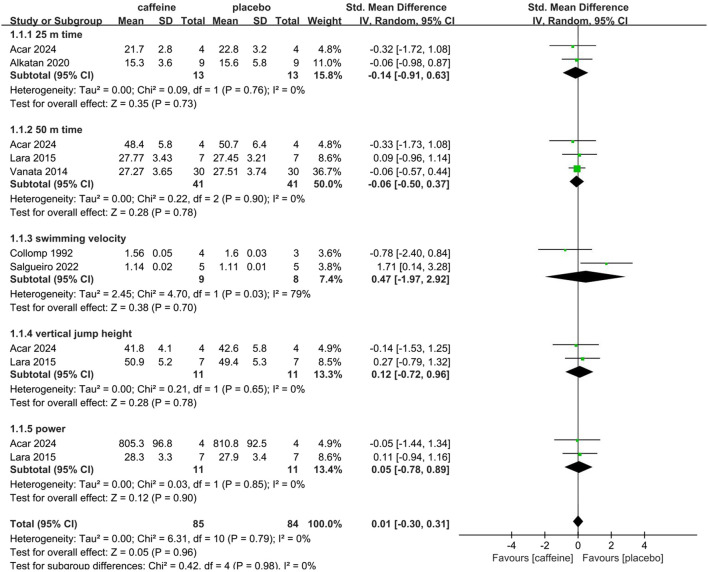
Subgroup analysis of caffeine’s effect on physical performance (25 m time, 50 m time, swimming velocity, jump height, power).

Six studies contributed to this analysis (Acar et al., 2024; Alkatan, 2020; Lara et al., 2015; Vanata et al., 2014; Collomp et al., 1992; Salgueiro et al., 2022). As shown in Figure 4, our SRMA indicated that caffeine had no overall effect on direct measures of physical performance (SMD: 0.01; 95% CI: -0.30, 0.31; p = 0.96; I^2^ = 0%). Subgroup analysis further showed a trivial but non-significant decrease in the 25 m time (SMD: -0.14; 95% CI: -0.91, 0.63; p = 0.73; I^2^ = 0%). Furthermore, the effect on 50 m time was non-significant (SMD: -0.06; 95% CI: -0.50, 0.37; p = 0.78; I^2^ = 0%). Additionally, swimming velocity showed no significant effect (SMD: 0.47; 95% CI: -1.97, 2.92; p = 0.70; I^2^ = 79%).

The impact of caffeine on jump height and power was evaluated in two studies (Acar et al., 2024; Lara et al., 2015). The results showed no advantage for the caffeine condition (SMD: 0.12, 95% CI: -0.72, 0.96; p = 0.78; I^2^ = 0%). Similarly, for power, the combined results also demonstrated no ergogenic benefit (SMD = 0.05; 95% CI: -0.78 to 0.89; p = 0.90; I^2^ = 0%).

#### 3.4.2 Physiological performance

**FIGURE 5:**
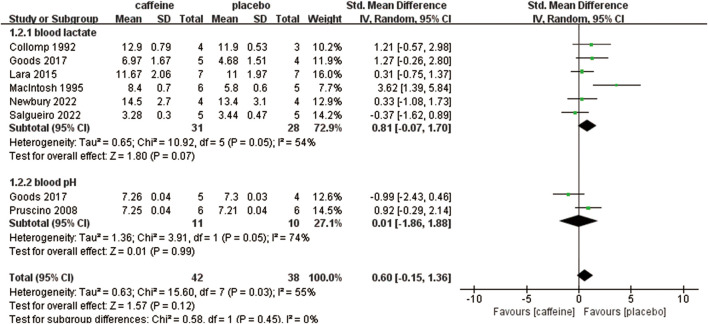
Subgroup analysis of caffeine’s effect on physiological performance (blood lactate, blood pH).

Seven studies evaluated the impact of caffeine on physiological performance (Collomp et al., 1992; Goods et al., 2017; Lara et al., 2015; MacIntosh and Wright, 1995; Newbury et al., 2022; Salgueiro et al., 2022; Pruscino et al., 2008). When compared to placebo, caffeine resulted in a moderate but non-significant increase in the overall effect (SMD: 0.60; 95% CI: −0.15, 1.36; p = 0.12; I^2^ = 55%). Subgroup analysis further revealed a large but non-significant enhancement in blood lactate (SMD: 0.81; 95% CI: −0.07, 1.70; p = 0.07; I^2^ = 54%). Likewise, a non-significant effect was observed for blood pH (SMD: 0.01; 95% CI: −1.86, 1.88; p = 0.99; I^2^ = 74%) (Figure 5).

## 4 Discussion

This study is the first SRMA to specifically examine both physical performance outcomes (e.g., 25 m time, 50 m time, swimming velocity, jump height, power) and physiological responses (e.g., blood lactate, blood pH) in swimmers. Our SRMA found no definitive evidence to support the notion that acute caffeine supplementation improves physical or physiological performance in swimmers. However, it is crucial to note that the evidence base is limited, with most outcomes being derived from only two studies. Additionally, some outcomes are associated with wide confidence intervals and high heterogeneity, which undermine the strength of the conclusions. Therefore, the current findings should be regarded as preliminary evidence, and further well-designed studies with larger sample sizes are needed to clarify the true effects of caffeine on swimmers’ performance.

### 4.1 Physical performance

Our SRMA suggests that acute caffeine supplementation does not significantly affect 25 m and 50 m swim times. This finding contrasts with the study by Grgic (2022) (Grgic, 2022), which identified a small yet statistically meaningful performance-enhancing impact of caffeine on short-distance swimming performance (25–75 m). Nevertheless, it should be emphasized that Grgic’s (2022) analysis combined performance data from 25 m, 50 m, and 75 m events without distinguishing between these distances. In contrast, our SRMA adopted a more detailed approach by separately analyzing 25 m and 50 m performances. This methodological distinction reduces the potential impact of physiological and metabolic differences across various swimming distances (Huang et al., 2024b), thereby increasing the specificity and interpretability of the results for different race formats. Nevertheless, similar to Grgic’s meta-analysis (Grgic, 2022), the current SRMA findings cannot yet support definitive conclusions. The limited number of studies available for each outcome means that even these separate analyses should be regarded as preliminary. Therefore, while our results suggests that caffeine supplementation may not reliably enhance sprint swimming performance, these findings should be considered provisional and interpreted with caution. Further research with larger sample sizes and more robust methodologies is required to strengthen the evidence base and provide more conclusive insights.

The finding that acute caffeine supplementation did not significantly improve 25 m swim performance aligns with the findings of Alkatan (2020) (Alkatan, 2020). In that study, a 250 mg caffeine dose did not significantly improve 25 m time. The authors suggested that caffeine’s ergogenic effects might be influenced by training status and individual variability. Specifically, individuals with lower training levels may not experience substantial benefits from caffeine, whereas elite athletes may exhibit heightened sensitivity to its effects due to superior acid-base regulation and neuromuscular efficiency. Additionally, individual differences in caffeine tolerance could modulate its impact on performance. However, contradictory findings are present in other studies. For example, Acar et al. (2024) reported that 6 mg/kg/bm of caffeine significantly improved 25 m swim times (Acar et al., 2024). Nonetheless, the current SRMA did not confirm this effect. Furthermore, the heterogeneity analysis (I^2^ = 0%) indicated high consistency among the included studies, suggesting that methodological variability is unlikely to explain the observed outcomes. A potential explanation for the discrepancy between individual studies and our SRMA is the limited statistical power. While some individual studies have reported significant performance improvements, the overall effect identified in our SRMA is constrained by the sample sizes of the included studies. Research on 25 m swimming is limited, and most studies have small sample sizes, which may hinder the detection of subtle ergogenic effects from caffeine supplementation. Furthermore, the standardized mean difference (SMD = −0.14) in our SRMA suggests that even if an effect exists, it is relatively small and may not lead to a practically meaningful improvement in overall performance. Therefore, caffeine intake may offer limited benefits for short-distance swimming performance, especially among trained athletes. Coaches and athletes should be cautious in expecting meaningful improvements in sprint events and consider individual variability in response. Sports nutritionists are advised to evaluate caffeine tolerance, training status, and event demands before making recommendations.

Our SRMA also did not confirm a significant improvement in 50 m time following acute caffeine supplementation. This finding contrasts with results from individual studies. For instance, Acar et al. (2024) reported a significant enhancement in 50 m swim performance after ingesting 6 mg/kg/bm of caffeine (Acar et al., 2024), a result consistent with Vanata et al. (2014) study (Vanata et al., 2014). Vanata et al. (2014) found that swimmers who consumed 3 mg/kg/bm of caffeine showed a significant improvement in 50-yard swim times, with 70% of participants demonstrating enhanced performance post-supplementation (Vanata et al., 2014). These findings suggest that caffeine may be beneficial for certain individuals under specific conditions. Several factors may account for the discrepancies between individual studies and our meta-analytic findings. First, caffeine’s ergogenic effect appears to be dose-dependent, with higher doses (e.g., ≥6 mg/kg/bm) potentially more effective than lower ones (e.g., 3 mg/kg/bm or 250 mg), which may be insufficient to elicit measurable performance gains (Vanata et al., 2014). Second, individual variability in caffeine metabolism, driven by genetic polymorphisms, sex-based differences, or habitual intake, can influence responsiveness (Vanata et al., 2014). Finally, it is worth noting that short-distance sprint swimming relies predominantly on anaerobic energy systems, whereas caffeine’s primary mechanisms (e.g., increased motor unit recruitment, CNS stimulation) may be more relevant to prolonged or aerobic efforts (Salgueiro et al., 2022). While individual variability, including training status, caffeine sensitivity, and genetic factors, may explain performance improvements observed in some trials, the pooled results from our meta-analysis indicate insufficient and inconsistent evidence to support a generalized ergogenic effect of caffeine on 50 m swim performance. Taken together, our findings suggest that while some swimmers may experience performance gains from caffeine ingestion under specific conditions, caffeine cannot be recommended as a universally effective ergogenic aid for 50 m swim performance. Coaches and athletes should carefully consider individual factors before incorporating caffeine into pre-competition strategies. In sprint swimming, where anaerobic demands dominate and marginal gains are critical, caffeine should be used selectively and with individualized assessment rather than as a standard supplement for all athletes.

Another explanation for the lack of significant effects of acute caffeine supplementation on 25 m and 50 m swim times is that its ergogenic benefits depend on time and fatigue, and may not fully manifest during short, isolated sprints. Caffeine’s main mechanisms, including central nervous system stimulation and enhanced motor unit recruitment, are more likely to contribute during prolonged or fatiguing exercise. In very short efforts dominated by anaerobic metabolism, these effects may be insufficient to yield measurable performance benefits (Astorino and Roberson, 2010). This may help explain why our pooled analysis of sprint swimming did not replicate the significant effects reported in some individual trials.

Our SRMA found no significant effect of caffeine supplementation on swimming velocity. This finding, however, should be interpreted with caution. On the one hand, only two studies contributed data to this outcome, limiting statistical power. On the other hand, the effect sizes varied notably in both magnitude and direction, which produced substantial heterogeneity (I^2^ = 79%). In addition, the wide confidence intervals suggest that the pooled estimate is unstable. Taken together, the available evidence is insufficient to establish a definitive conclusion regarding caffeine’s impact on swimming speed. Further trials with larger samples and more standardized designs are warranted to clarify whether caffeine can consistently influence swimming velocity across different populations and exercise conditions.

This study is the first SRMA to quantify the effects of caffeine on jump height and power in swimmers. Our SRMA suggests that consuming caffeine failed to produce a significant improvement in jump height and power. Jump height serves as a crucial indicator of lower-limb muscle power, which is particularly vital in short-distance swimming (Girold et al., 2012). The quality of the start directly influences water entry speed, and takeoff performance is closely linked to lower-limb explosiveness (Markovic and Jaric, 2007). Although the overall analysis did not establish a significant effect of caffeine on jump performance, the results of the two included studies (Acar et al., 2024; Lara et al., 2015) were not entirely consistent. For instance, Acar et al. (2024) reported that after ingesting 6 mg/kg/bm of caffeine, female swimmers exhibited increased neural excitability but no significant improvement in vertical jump height. The authors suggested that this may be due to the nature of swimming training, which primarily emphasizes upper-body strength and aerobic endurance, with lower-limb propulsion contributing only 12% to overall swimming performance. Consequently, swimmers may exhibit relatively lower jump performance, which could explain the absence of a significant response to caffeine supplementation (Acar et al., 2024). In contrast, Lara et al. (2015) found that ingesting 3 mg/kg/bm of caffeine significantly improved jump height in short-distance swimmers (Lara et al., 2015). This discrepancy may be attributable to the differences in caffeine dosage (3 mg/kg/bm vs. 6 mg/kg/bm). A study indicates that higher doses of caffeine may induce anxiety or excessive sympathetic nervous system activation in certain individuals (Guest et al., 2021), potentially negating its benefits for explosive movements (Astorino et al., 2008). Our SRMA also examined the effects of caffeine on power output. Both included studies reported no significant ergogenic effect on power (Acar et al., 2024; Lara et al., 2015). This lack of improvement may be explained by the limited emphasis on explosive lower-limb training in swimming, where propulsion is predominantly generated by the upper body (Aujouannet et al., 2006). Moreover, the assessment methods used to measure power in dryland settings may not fully capture the transferability of caffeine’s potential benefits to sport-specific swimming actions such as starts and turns (Potdevin et al., 2011). Although caffeine is known to increase neuromuscular drive and motor unit recruitment (Astorino and Roberson, 2010), these effects may be too transient to produce measurable gains in peak power during very short explosive efforts. Future studies should therefore adopt more sensitive and sport-specific measures of power, and investigate whether caffeine’s influence on lower-limb power can contribute meaningfully to key performance phases in swimming. It is important to note, however, that only two studies were available for inclusion, both with relatively small sample sizes. Therefore, these findings should be regarded as preliminary. Future research should prioritize large-scale, sex-balanced randomized controlled trials, employ stratified designs to differentiate responders from non-responders, and further explore the relationship between caffeine, lower-limb explosiveness, and sport-specific performance. Such studies will be essential to clarify the true role of caffeine in swimming and to provide more robust evidence for practice.

While some individual studies reported significant ergogenic effects of caffeine on physical performance, the pooled analysis did not show statistical significance. Several methodological factors may explain this difference. First, the available evidence is inconsistent, and our inclusion criteria required both significant and non-significant results. Second, variations across studies in race distance, caffeine dose, timing of ingestion, and training level of participants may have influenced the outcomes. Although we attempted to reduce confounding by analyzing 25 m and 50 m events separately, the number of studies for each outcome was still limited. Third, the small number of studies and their modest sample sizes reduced the statistical power, leading to wide confidence intervals that included zero. Finally, the observed heterogeneity (e.g., I^2^ = 79% for swimming velocity) suggests that the true effect of caffeine may depend on specific populations and protocols. Together, these factors help explain why the pooled analysis did not confirm the significant findings of some individual trials and indicate that the current results should be considered preliminary and interpreted with caution. Future studies with larger sample sizes, standardized caffeine protocols, and stratified analyses (e.g., by training status, sex, or genetic variability) are needed to clarify the true impact of caffeine on swimming performance.

Despite these limitations, this study has methodological value. Unlike previous reviews, this SRMA is the first to distinguish between 25 m and 50 m performances and to include jump height and power as indirect physical outcomes. This approach improves the specificity and interpretability of the results and expands the evidence base. Therefore, although the conclusions are not yet definitive, this work provides a foundation for future large, well-designed studies and offers useful guidance for clarifying the role of caffeine in different aspects of swimming performance.

### 4.2 Physiological performance

Blood lactate is a marker of anaerobic metabolism (Gladden, 2004; Brooks, 2001; Beneke et al., 2011), while blood pH reflects acid–base balance; both parameters are closely related during high-intensity exercise (Robergs et al., 2004). This SRMA comprehensively evaluated the effects of acute caffeine supplementation on blood lactate and blood pH levels in swimmers. The results indicated that caffeine intake did not induce significant overall changes in blood lactate, supporting the findings of Newbury et al. (2022), who observed no substantial effect of caffeine on blood lactate concentration, despite a slight increase compared to placebo within 60 min post-ingestion (Newbury et al., 2022). However, conflicting results have been reported. Collomp et al. (1992) documented a significant rise in blood lactate levels following caffeine ingestion, observed in both trained and untrained individuals. Notably, only the trained participants demonstrated improved swimming speed, whereas untrained individuals showed no measurable enhancement. The authors suggested that this discrepancy may be due to differences in muscle buffering capacity; trained athletes possess a greater ability to manage hydrogen ion (H^+^) accumulation, thereby mitigating the effects of metabolic acidosis. This enhanced buffering capacity may allow trained swimmers to better utilize caffeine-induced metabolic changes to improve performance (Collomp et al., 1992). In contrast, Salgueiro et al. (2022) observed a reduction in blood lactate levels following ingestion of 6 mg/kg/bm of caffeine during high-intensity interval training, particularly in the later stages of repeated sprint sessions (Salgueiro et al., 2022). The authors speculated that caffeine might enhance aerobic metabolism, reducing reliance on anaerobic pathways and thus decreasing lactate production. While current evidence is limited and mixed, these findings suggest that caffeine’s influence on metabolic responses may depend on training status and exercise modality. In practice, coaches and sports practitioners should avoid relying on blood lactate changes as a direct indicator of caffeine’s ergogenic potential in swimmers, and instead evaluate performance outcomes holistically, considering individual physiological profiles.

Regarding blood pH, our SRMA results indicate that acute caffeine intake does not improve this parameter. However, several studies have indicated that caffeine may exacerbate metabolic acidosis. For instance, Goods et al. (2017) observed that during a 6 × 75 m swimming sprint test, caffeine not only increased blood lactate levels but also significantly decreased blood pH, with this trend persisting throughout the sprint session. The authors proposed that this effect might result from caffeine-induced glycolysis, which accelerates hydrogen ion (H^+^) accumulation, thereby exacerbating metabolic acidosis (Goods et al., 2017). Similarly, Pruscino et al. (2008) reported that ingestion of 6.2 ± 0.3 mg/kg/bm of caffeine led to a greater post-exercise decline in blood pH and a slower recovery rate, indicating that caffeine might increase acid load and hinder post-exercise recovery. However, the study also found that co-ingestion of sodium bicarbonate with caffeine attenuated the decline in blood pH, suggesting that additional buffering agents may mitigate caffeine-induced acidosis (Pruscino et al., 2008). The inconsistencies across these findings may be attributed to several factors, including athletes’ training status, exercise modalities, caffeine dosage, and individual metabolic variability. For example, Collomp et al. (1992) proposed that training status could influence an individual’s tolerance to acid-base imbalances, with trained athletes exhibiting greater buffering capacity, allowing them to better adapt to fluctuations in blood lactate levels. In contrast, untrained individuals may experience more pronounced performance impairments due to exacerbated acidosis (Collomp et al., 1992). Additionally, variations in exercise modalities could lead to distinct metabolic responses. For instance, Goods et al. (2017) employed a repeated sprint test, which relies heavily on anaerobic metabolism (Goods et al., 2017), while Salgueiro et al. (2022) utilized high-intensity interval training, which permits a greater aerobic contribution (Salgueiro et al., 2022). Moreover, differences in caffeine dosages across studies could have influenced the results. Some research suggests that higher doses of caffeine may enhance anaerobic metabolism (MacIntosh and Wright, 1995), but in certain individuals, high caffeine intake may also induce anxiety or excessive sympathetic activation (Guest et al., 2021; Astorino et al., 2008), potentially altering metabolic responses. Although based on limited evidence, our SRMA indicates that acute caffeine intake does not significantly affect blood pH. However, given that some studies have reported caffeine-induced acidosis during high-intensity efforts, athletes and coaches should remain cautious. Caffeine use should be individualized and, when necessary, paired with buffering strategies such as sodium bicarbonate to minimize potential acid–base disturbances.

The interpretation of these pooled results is constrained by the relatively high heterogeneity observed in both blood lactate (I^2^ = 54%) and blood pH (I^2^ = 74%). Such heterogeneity indicates that the effects of caffeine on these physiological outcomes are inconsistent across studies and may depend on specific factors such as exercise modality, training status, or supplementation protocol. Consequently, the absence of a statistically significant pooled effect does not necessarily imply that caffeine has no impact, but rather that variability between studies prevents a clear and consistent conclusion. Future investigations should employ standardized methodologies, larger and more stratified samples, and greater consistency in the measurement of physiological outcomes to reduce heterogeneity and improve interpretability.

While some individual studies reported significant changes in physiological outcomes after caffeine ingestion, the pooled analysis did not reach statistical significance. This discrepancy can be explained by several methodological factors. First, the available evidence is inherently inconsistent, and our inclusion criteria required both significant and non-significant trials. Second, variations across studies in caffeine dosage, timing of ingestion, exercise modality, and training status of participants may have influenced the outcomes. Third, the small number of studies and their modest sample sizes limited statistical power, resulting in wide confidence intervals that included zero. Together, these factors help explain why the pooled analysis did not replicate the significant effects observed in some individual studies and indicate that the current findings should be regarded as preliminary and interpreted with caution. Importantly, this SRMA is the first to systematically include physiological indicators such as blood lactate and blood pH, thereby expanding the scope of available evidence and providing a more comprehensive foundation for future research into the combined effects of caffeine on metabolism and performance.

### 4.3 Limitations and strengths

A major strength of this study lies in its comprehensive, distance-specific analysis, which separately evaluates 25 m and 50 m swim performance. Unlike previous meta-analyses that combined multiple sprint distances, this approach enhances the specificity of the findings. Furthermore, this SRMA is the first to evaluate caffeine’s effects on both jump height, power and physiological parameters in swimmers, expanding the evidence base regarding its multifaceted effects, despite the lack of observed ergogenic benefits.

However, several constraints should be acknowledged. First, the limited number of available studies for each outcome, with some analyses based on only two trials, substantially reduced the statistical power of the pooled estimates. This means that while the meta-analysis provides a systematic synthesis of the available evidence, the conclusions must be regarded as preliminary and interpreted with caution. The small sample sizes of individual studies further compounded this limitation. Furthermore, the small sample sizes in individual studies compounded this limitation. Therefore, future research should prioritize larger-scale randomized controlled trials to strengthen the robustness of the findings. Additionally, variability in caffeine dosage and supplementation protocols across studies may have influenced the consistency of the results. The timing of caffeine ingestion relative to exercise testing also varied, potentially affecting its physiological effects. Ideally, factors such as dosage, ingestion timing, training status, sex, and genetic variability should be examined as potential moderators of caffeine’s ergogenic effects; however, the small number of available studies prevented reliable subgroup analyses or meta-regressions in the present SRMA. Future studies should standardize caffeine dosage and supplementation timing to improve the comparability of results. Moreover, the studies included in this meta-analysis did not consistently report or categorize participants by training level, age, or sex, which are factors that may influence athletic performance and the response to caffeine supplementation. Although we included all swimmers regardless of their training level (e.g., high school vs. collegiate athletes), future research should consider categorizing athletes by training level to better understand the differential effects of caffeine in these subgroups. Age and sex could also serve as important moderators of caffeine’s ergogenic effects, and their potential roles should be systematically investigated in future studies. Finally, while this study focused on the short-term effects of acute caffeine supplementation in swimmers, the long-term impact on performance and training adaptations remains uncertain. Whether prolonged caffeine intake influences training adaptations, recovery capacity, and overall performance requires further investigation.

## 5 Conclusion

This SRMA found no significant evidence that acute caffeine supplementation improves physical or physiological performance in swimmers. Given that several outcomes were based on only two small studies and some results showed high heterogeneity, the findings should be regarded as preliminary and interpreted with caution. Future research should include larger and sex-balanced randomized controlled trials with standardized outcome definitions, consistent measurement time points, and stratified designs that distinguish responders from non-responders. Considering moderating factors such as training status, caffeine tolerance, and genetic variability will also be essential to clarify caffeine’s true effects on swimming performance and to provide stronger evidence for practical recommendations.

## Data Availability

The original contributions presented in the study are included in the article/Supplementary Material, further inquiries can be directed to the corresponding author.

## Abbreviations

IOC, International Olympic Committee; ISSN, International Society of Sports Nutrition; SRMA, Systematic Review and Meta-analysis; PEDro, Physiotherapy Evidence Database; SMD, Standardized Mean Differences; CI, Confidence Intervals.
